# Supplementary material for: Prognostic Role of Lung Ultrasound in Children with Bronchiolitis: Multicentric Prospective Study
Source: J Clin Med. 2022 Jul 21;11(14):4233. doi: 10.3390/jcm11144233 (PMC9316238; doi:10.3390/jcm11144233)
Supplement: Supplementary file 1 [file jcm-11-04233-s001.zip › jcm-1749927-supplementary.pdf]

**SUPPLEMENTARY MATERIAL**

**Supplementary Figure S1. Schematic classification of artifacts. We have classified artifacts into seven types.** A-Lines identify the normal finding. A-lines are hyperechoic lines with run more or less horizontally across the ultrasound screen as replicas of the pleural line. They represent the normal pattern of the lung. The Short Vertical Artifacts (SVA) are vertical artifacts that lose their brightness before reaching the bottom of the screen, showing more or less rapid fading. They were not considered significant.

B-lines are defined as hyperechoic artifacts which originate at the pleura line and lie roughly perpendicular to the latter. Isolated B lines were not considered significant. Multiple B-lines are many B-lines in each scan. Multiple B lines were considered pathological findings. The white lung are completely white echo graphic lung field and no horizontal reverberation. It was considered an independent pathological finding. Consolidations were classified according to their linear size (greater superficial extension  consolidation = < 1 cm or > 1 cm).

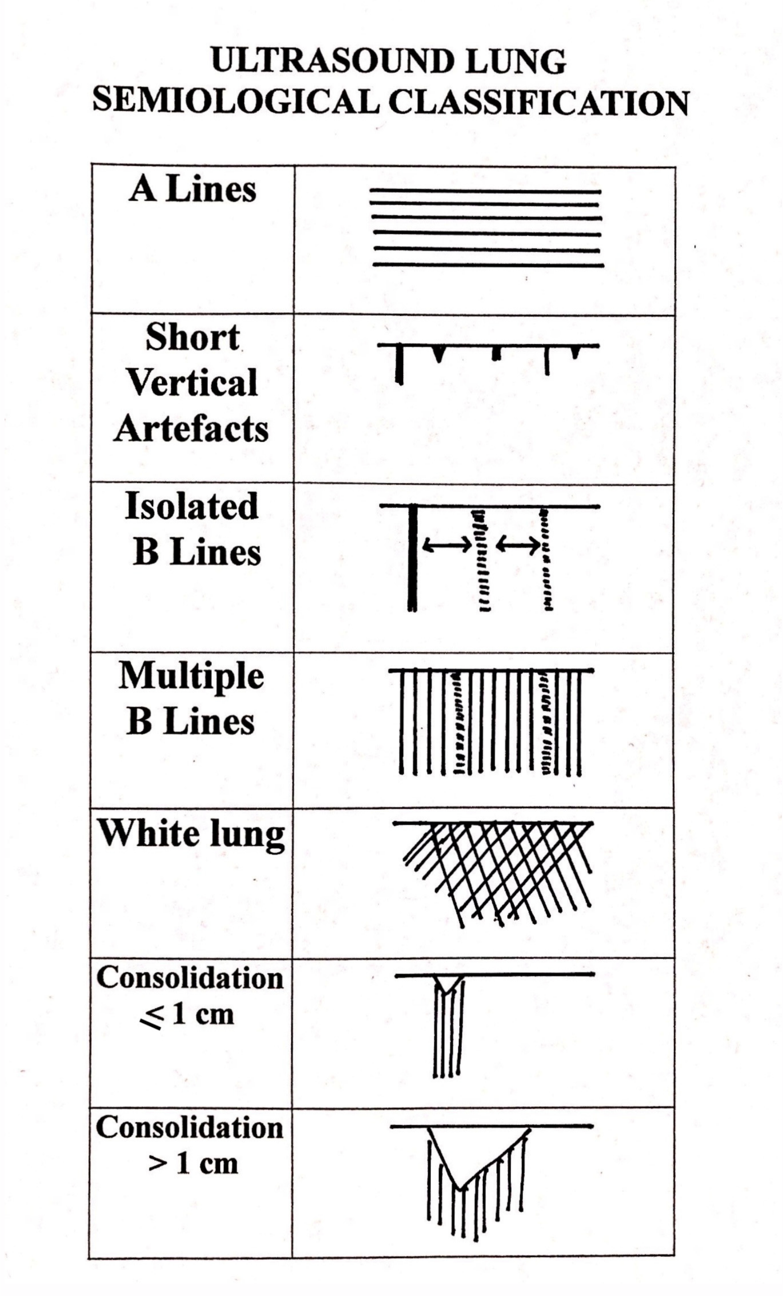

| | ITALY CENTERS PARTECIPATING IN THE STUDY | Ultrasound Machine Used | CASES with 4 controls | CASES with a check | Excluded | Total cases | TOTAL CASES ENROLLED |
|---|---|---|---|---|---|---|---|
| **1** | Pediatric Unit, Valle del Serchio General Hospital, Barga, Lucca | Esaote My lab 50 | 19 | 11 | 0 | 30 | 30 |
| **2** | Division of Pediatric and Neonatology Unit. Cecina Civil Hospital; Cecina, Livorno | Esaote My Lab six | 28 | 1 | 4 | 33 | 29 |
| **3** | Pediatric Unit, Moriggia Pelascini Hospital, Gravedona et Uniti, Como | Esaote My Lab Alpha | 31 | 7 | 6 | 44 | 38 |
| **4** | Department of Pediatrics, Lodi Hospital, Lodi | Aloka alfa 10, Vinno 6, Esaote Mylab | 3 | 0 | 0 | 3 | 3 |
| **5** | Unit of Pediatrics and Pediatric Emergency. Catania | **Esaote Mylab Alpha** | 0 | 1 | 0 | 1 | 1 |
| **6** | Division of Neonatology And Neonatal Intensive Care Unit. Pisa. | GE Vivid Iq | 3 | 3 | 2 | 8 | 6 |
| **7** | Department of Woman and Child Health and Public Health, Fondazione Policlinico Universitario A. Gemelli IRCCS, Rome | Esaote My lab40 | 44 | 8 | 1 | 53 | 52 |
| **8** | Department of Pediatric Emergency, Bambin Gesù Children's Hospital IRCCS, Rome | Sonosite EDGE II | 23 | 8 | 0 | 31 | 31 |
| **9** | Pediatric and Pediatric Emergency Room Unit, Cannizzaro Emergency Hospital - Catania | GE LogiQ, TWADP | 11 | 7 | 5 | 23 | 18 |
| **10** | Pediatric Emergency Unit, Sant'Orsola Hospital IRCCS, Bologna | Mindray DCT6 | 7 | 1 | 0 | 8 | 8 |

| | ITALY CENTERS PARTECIPATING IN THE STUDY | Ultrasound Machine Used | CASES with 4 controls | CASES with a check | Excluded | Total cases | TOTAL CASES ENROLLED |
|---|---|---|---|---|---|---|---|
| **11** | Division of Pediatric and Neonatology Unit. Nottola Hospital, Montepulciano, Siena | Toshiba Xario 200 | 8 | 0 | 1 | 9 | 8 |
| **12** | Pediatric Clinic, Department Of Surgical and Biomedical Sciences, University of Perugia, Perugia | Philips HD15, GE Vivid E95 | 8 | 1 | 0 | 9 | 9 |
| **Tot.** | | | 185 | 48 | 19 | 252 | 233 |

**Supplemental Table S1.** Italian centers participating in the study. 12 Italian centers with different degrees of intensity of care (1,2,3,level) participated in the study.

| DICOTHOMOUS QUALITATIVE CLASSIFICATION: Positive ultrasound vs negative ultrasound (MODEL 1) | |
|---|---|
| **Negative ultrasound** | All 8 lung fields of an ultrasound are normal |
| **Positive ultrasound** | At least 1 lung field of 8 is not normal |
| **DICOTHOMOUS QUALITATIVE CLASSIFICATION: Positive ultrasound vs negative ultrasound (MODEL 1)** | |
| **Negative field** | Isolated or combined artifacts are present: A-SVA-IB |
| **Positive field** | There is at least one of the following artifacts: MB, WL, C, CC |
| **QUALITATIVE OUTCOME OF THE ULTRASOUND (MODEL 2)** | |
| **Normal ultrasound** | If all 8 fields are normal |
| **Interstitial ultrasound** | If at least 1 in 8 field is interstitial and the other normal |
| **Consolidative ultrasound** | If at least 1 field out of 8 is consolidative and the other normal |
| **Mixed ultrasound** | If present together an interstitial and/or consolidative and/or mixed field |
| **QUALITATIVE OUTCOME OF THE ULTRASOUND (MODEL 2)** | |
| **Normal field** | If isolated or combined artifacts are present: A-SVA-IB |
| **Interstitial field** | If any isolated or combined artifacts are present: MB-WL |
| **Consolidative field** | If isolated or combined artifacts are present: C-CC |
| **Mixed field** | If isolated or combined artifacts are present: MB-WL-C-CC |
| **QUANTITIVE LUS score 0-32** | |
| **Ultrasound signs** | SCORE GIVEN TO ULTRASOUND SIGNS |
| A lines | 0 |
| Short Vertical artifacts | 1 |
| Isolate B Lines | 1 |
| Multiple B Lines | 2 |
| White Lung | 3 |
| Consolidation =< 1cm | 3 |

| | |
|---|---|
| Consolidation > 1 cm | 4 |
| | For each ultrasound field explored in case of association of artifacts is the artifact with the highest score (e.g. WL+ IB= 3 and not 4) |
| **QUANTITIVE LUS score with cut off = 9** | |
| **Ultrasound score < = 9** | Mild ultrasound |
| **Ultrasound score > 9** | Moderate-severe ultrasound |

**Supplementary Table S2 Definitions of adopted 2 qualitative models and 2 quantitative models score. ABBREVIATIONS:** A: A lines; SVA: Short Vertical Artifacts; IB: Isolated B Lines; MB: Multiple B Lines; WL: White Lung; C: Consolidation = < 1 cm; CC: Consolidation > 1 cm.

**Supplementary Table S3. The comparison between the AUC of the 2 qualitative models without and with the other LUS evidences**

| Models AUC comparisons | AUC Difference | p-value |
|---|---|---|
| Qualitative LUS model (positive/negative) - Qualitative LUS model (4-level model) | -0,054 | 0,027 |
| Qualitative LUS model (positive/negative) - Quantitative LUS model (score) | -0,161 | <0,001 |
| Qualitative LUS model (positive/negative) - Quantitative LUS model (cutoff score >9) | -0,131 | <0,001 |
| Qualitative LUS model (positive/negative) - Qualitative LUS model (positive/negative)** | -0,138 | <0,001 |
| Qualitative LUS model (positive/negative) - Qualitative LUS model (4-level model)** | -0,144 | <0,001 |
| Qualitative LUS model (positive/negative) - Quantitative LUS model (score)** | -0,165 | <0,001 |
| Qualitative LUS model (positive/negative) - Quantitative LUS model (cutoff score >9)** | -0,156 | <0,001 |
| Qualitative LUS model (4-level model) - Quantitative LUS model (score) | -0,107 | <0,001 |
| Qualitative LUS model (4-level model) - Quantitative LUS model (cutoff score >9) | -0,076 | 0,006 |
| Qualitative LUS model (4-level model) - Qualitative LUS model (positive/negative)** | -0,084 | 0,001 |
| Qualitative LUS model (4-level model) - Qualitative LUS model (4-level model)** | -0,089 | 0,001 |
| Qualitative LUS model (4-level model) - Quantitative LUS model (score)** | -0,111 | <0,001 |
| Qualitative LUS model (4-level model) - Quantitative LUS model (cutoff score >9)** | -0,101 | <0,001 |
| Quantitative LUS model (score) - Quantitative LUS model (cutoff score >9) | 0,030 | 0,125 |
| Quantitative LUS model (score) - Qualitative LUS model (positive/negative)** | 0,023 | 0,158 |
| Quantitative LUS model (score) - Qualitative LUS model (4-level model)** | 0,017 | 0,264 |
| Quantitative LUS model (score) - Quantitative LUS model (score)** | -0,004 | 0,377 |
| Quantitative LUS model (score) - Quantitative LUS model (cutoff score >9)** | 0,005 | 0,641 |
| Quantitative LUS model (cutoff score >9) - Qualitative LUS model (positive/negative)** | -0,007 | 0,739 |
| Quantitative LUS model (cutoff score >9) - Qualitative LUS model (4-level model)** | -0,013 | 0,557 |
| Quantitative LUS model (cutoff score >9) - Quantitative LUS model (score)** | -0,035 | 0,079 |
| Quantitative LUS model (cutoff score >9) - Quantitative LUS model (cutoff score >9)** | -0,025 | 0,150 |
| Qualitative LUS model (positive/negative)** - Qualitative LUS model (4-level model)** | -0,006 | 0,294 |
| Qualitative LUS model (positive/negative)** - Quantitative LUS model (score)** | -0,027 | 0,115 |
| Qualitative LUS model (positive/negative)** - Quantitative LUS model (cutoff score >9)** | -0,018 | 0,027 |
| Qualitative LUS model (4-level model)** - Quantitative LUS model (score)** | -0,022 | 0,199 |
| Qualitative LUS model (4-level model)** - Quantitative LUS model (cutoff score >9)** | -0,012 | 0,147 |
| Quantitative LUS model (score)** - Quantitative LUS model (cutoff score >9)** | 0,010 | 0,466 |

| LUNG ULTRASOUND MODELS | | All cases n= 216 | RSV positive n= 126 | RSV negative n= 90 | P values |
|---|---|---|---|---|---|
| **Ultrasound positive vs negative** | **Ultrasound positive, n° (%)** | 170 (78.7) | 101 (80.2) | 69 (91,8) | 0.537 |
| | **Ultrasound negative, n° (%)** | 46 (21.3) | 25 (19.8) | 21 (23.3) | |
| **Qualitative result ultrasound** | **Normal, n° (%)** | 46 (21.3) | 25 (19.8) | 21 (23.3) | 0.888 |
| | **Interstitial, n° (%)** | 15 (6.9) | 9 (7.1) | 6 (6.7) | |
| | **Consolidative, n° (%)** | 53 (24.6) | 30 (23.8) | 23 (25.6) | |
| | **Mixed n° (%)** | 102 (47.2) | 62 (49.3) | 40 (44.44) | |
| **Score, mean** (SD) | | 9.6 (5.2) | 10.1 (5.6) | 8.7 (4.5) | 0.041 |
| **Score cut off > 9,** n (%) | Yes, n° (%) | 99 (45.8%) | 68 (54.0%) | 31 (34.4%) | 0.005 |
| | No, n° (%) | 117 (54.2%) | 58 (46.0%) | 59 (65.6%) | |

**Supplementary Table S4** Comparison between bronchiolitis VRS +/.

| Model | Variable | | OR | 95% CI | | P value |
|---|---|---|---|---|---|---|
| Univariate models | **Positive ultrasound** | Yes | 2,082 | 1,617 | 2,679 | 0,001 |
| | | No | 1 | | | |
| | **Ultrasound result** | Interstizial | 1,950 | 1,496 | 2,542 | 0,001 |
| | | Consolidative | 1,811 | 1,402 | 2,339 | 0,001 |
| | | Mixed | 2,047 | 1,602 | 2,616 | 0,001 |
| | | Normal | 1 | | | |
| | **Score** | | 1,117 | 1,087 | 1,148 | 0,001 |
| | **Score > 9** | Yes | 3,291 | 2,467 | 4,391 | 0,001 |
| | | No | 1 | | | |
| **MULTIVARIATE ANALYSES USING DIFFERNT LUS MODELS** | **MULTIVARIATE MODELS BASED ON QUALITATIVE LUS (positive vs negative) without or with other LUS variables (Early involvement of paravertebral lung fields and extension)** | | | | | |
| | **Positive ultrasound** | Yes | 1,945 | 1,054 | 3,587 | 0,033 |
| | | No | 1 | | | |
| | **Early involvement of lung fields** | Yes | 2,609 | 1,316 | 5,172 | 0,006 |
| | | No | 1 | | | |
| | **Number of fields involved** | | 1,285 | 1,167 | 1,416 | 0,000 |
| **MULTIVARIATE ANALYSES USING DIFFERNT LUS MODELS** | **MULTIVARIATE MODELS BASED ON QUALITATIVE LUS (type of lung disease) without or with other LUS variables (Early involvement of paravertebral lung fields and extension)** | | | | | |
| | **Ultrasound result** | Interstizial | 2,066 | 1,077 | 3,962 | 0,029 |
| | | Consolidative | 1,850 | 0,982 | 3,483 | 0,057 |

| | | | | | |
|---|---|---|---|---|---|
| | Mixed | 1,906 | 1,003 | 3,624 | 0,049 |
| | Normal | 1 | | | |
| **Early involvement of lung fields** | Yes | 2,704 | 1,364 | 5,362 | 0,004 |
| | No | 1 | | | |
| **Number of fields involved** | | 1,271 | 1,150 | 1,403 | 0,000 |
| **MULTIVARIATE MODELS BASED ON QUANTITATIVE LUS (mean score) without or with other LUS variables (Early involvement of paravertebral lung fields and extension)** | | | | | |
| **Score** | | 1,119 | 1,085 | 1,153 | 0,000 |
| **Early involvement of lung fields** | Yes | 2,444<br>2 | 1,245 | 4,788 | 0,009 |
| | No | 1 | | | |
| **MULTIVARIATE MODELS BASED ON QUANTITATIVE LUS (score >9) without or with other LUS variables (Early involvement of paravertebral lung fields and extension)** | | | | | |
| **Score >9** | Yes | 2,605 | 1,694 | 4,005 | 0,000 |
| | No | 1 | | | |
| **Early involvement of lung fields** | Yes | 2,366 | 1,177 | 4,755 | 0,016 |
| | No | 1 | | | |
| **Number of fields involved** | | 1,158 | 1,042 | 1,286 | 0,006 |

**Table S5.** Multivariate analyses correlating LUS and bronchiolitis severity at last follow-up.

| | | TO | | | T1 | | | T2 | | | T3 | | | P Value |
|---|---|---|---|---|---|---|---|---|---|---|---|---|---|---|
| | | **CASES (n=185)** | **Mild bronchilitis (n=129),69,7 %** | **Moderate/severe Bronchiolitis (n=56), 30,3%** | **CASI (n=185)** | **Mild bronchilitis (n=136), 73,5%** | **Moderate/severe Bronchiolitis (n=49), 26,5%** | **CASES (n=185)** | **Mild Bronchiolitis (n=152), 82,2%** | **Moderate/severe Bronchiolitis (n=33), 17,8%** | **CASES (n=185)** | **Mild bronchilitis (n=177), 95,7%** | **Moderate/severe Bronchiolitis (n=8), 4,3%** | **0,000** |
| THERAPIES | **Oxygen** | 62 (33,5%) | 19 (14,7%) | 43 (76,8%) | 71 (38,4%) | 25 (18,4%) | 46 (93,9%) | 71(0,384%) | 43 (0,283%) | 28 (0,848%) | 39 (0,211%) | 31 (0,175%) | 8 (1%) | 0,005 |
| | **Antibiotics** | 62 (33,5%) | 36(27,9%) | 26 (46,4%) | 68 (36,8%) | 40 (29,4%) | 26 (57,1%) | 69 (0,373%) | 48 (0,316%) | 21 (0,636%) | 57 (0,308%) | 57 (0,294%) | 5 (0,625%) | 0,441 |
| | **Hypertonic solucion** | 80 (43,2%) | 57 (44,2%) | 23 (41,1%) | 77 (41,6%) | 58 (46,2%) | 19 (38,8%) | 79 (0,472%) | 62 (0,408%) | 17 (0,515%) | 71 (0,384%) | 68 (0,384%) | 3 (0,375%) | 0,022 |
| T | **Broncodilators** | 68 (36,8%) | 41 (31,8%) | 27 (48,2%) | 70 (37,8%) | 47 (34,6%) | 23 (46,9%) | 74 (40%) | 62 (40,8%) | 12 (36,4%) | 57 (30,8%) | 51 (28,8%) | 6 (75%) | 0,035 |
| | **Steroids** | 73 (39,5%) | 42 (32,6%) | 31 (55,4%) | 73 (39,5%) | 47 (34,6%) | 26 (53,1%) | 73 (39,5%) | 59 (38,8%) | 14 (42,4 %) | 53 (28,6%) | 47 (26,6%) | 6 (75%) | 0,002 |
| | **Adrenaline** | 3 (1,6%) | 0 | 3 (5,4%) | 3 (1,6%) | 0 | 3 (6,1%) | 2 (11%) | 0 | 2 (6,1%) | 1 (0,5%) | 1 (0,6%) | 0 | 0,625 |
| | **HFNC** | 37 (20%) | 12 (9,3%) | 35 (44,6%) | 39 (21,1%) | 16 (11,8%) | 23 (46,9%) | 29 (15,7%) | 16 (10,5) | 13 (39,4%) | 13 (7%) | 9 (5,1%) | 4 (50%) | < 0,001 |
| | **nCPAP** | 2 (1,1%) | 0 ( | 2 (3,6%) | 7 (3,8%) | 0 | 7 (14,3%) | 7 (3,8%) | 4 (2,6%) | 3 (9,1%) | 2 (1,1%) | 1 (0,6%) | 1 (12,5 %) | 1 |
| | **Invasive Ventilation** | 0 | 0 | 0 | 0 | 0 | 0 | 0 | 0 | 0 | 0 | 0 | 0 | |

**Table S6** Changes in treatments offered. HFNC: high flow nasal cannulae. nCPAP: nasal Continuous Positive Airway Preassure
